# Bacterial nomenclature in the era of genomics

**DOI:** 10.1016/j.nmni.2021.100942

**Published:** 2021-09-25

**Authors:** M.J. Pallen

**Affiliations:** 1) Quadram Institute Bioscience, Norwich Research Park, Norwich, UK; 2) Norwich Medical School, University of East Anglia, Norwich Research Park, Norwich, UK; 3) School of Veterinary Medicine, University of Surrey, Guildford, Surrey, UK

**Keywords:** Bacterial nomenclature, bacterial taxonomy, bioinformatics, metagenomics, microbial genomics

## Abstract

The remarkable success of taxonomic discovery, powered by culturomics, genomics and metagenomics, creates a pressing need for new bacterial names while holding a mirror up to the slow pace of change in bacterial nomenclature. Here, I take a fresh look at bacterial nomenclature, exploring how we might create a system fit for the age of genomics, playing to the strengths of current practice while minimizing difficulties. Adoption of linguistic pragmatism—obeying the rules while treating recommendations as merely optional—will make it easier to create names derived from descriptions, from people or places or even arbitrarily. Simpler protologues and a relaxed approach to recommendations will also remove much of the need for expert linguistic quality control. Automated computer-based approaches will allow names to be created en masse before they are needed while also relieving microbiologists of the need for competence in Latin. The result will be a system that is accessible, inclusive and digital, while also fully capable of naming the unnamed millions of bacteria.

## Highlights

• There is a need for new names, but most microbiologists lack competence in Latin.
• Digital approaches to creation of names will minimize the need for human expertise.
• We can easily create descriptive and arbitrary Latinized names en masse.
• Linguistic pragmatism will make bacterial nomenclature accessible and inclusive.
• Simpler protologues will make publishing names easier and less intimidating.

## Introduction

We, microbiologists, live in exciting times. The ready availability of genome sequences has transformed our discipline, fuelling exponential growth in the identification of new species while also enabling comprehensive sequence-based taxonomies, such as the Genome Taxonomy Database (GTDB) [[1], [2], [3]]. This exhilarating success creates an urgent new challenge: how are we going to name all these new species? In addition, fast-moving progress in taxonomic discovery holds a mirror up to the slow pace of change in bacterial nomenclature, where the relevant code (the International Code for Nomenclature of Prokaryotes, the ICNP or ‘the Code’) was last updated over a decade ago [4] and contains many statements that have remained largely unchanged since the 1860s [5]. A revision to the ICNP is expected in 2022 [6]. However, aside from incorporating *Cyanobacteria* and the rank of phylum [7,8], most proposed edits simply tinker with minutiae, while the most significant proposed change in recent years—the much-discussed use of sequence as type [9,10] —has been rejected [11]. As a result, work is afoot to establish a new code, the SeqCode, to run in parallel to the ICNP while extending priority to names that use genome sequences as type material [12].

Here, I take a fresh look at bacterial nomenclature, re-evaluating the strengths and weakness of the current system, before exploring how we might create a nomenclature fit for the age of genomics—playing to the strengths of the ICNP while avoiding current difficulties in practice. I will restrict discussion to linguistic issues, avoiding arguments as to how taxa are described or tied to type material or how names are handled when taxonomies change. For simplicity, I will generally use the terms ‘bacteria’ and ‘bacterial’, although what I have to say is pertinent to naming Archaea. I will avoid the term ‘prokaryote’, which has no place in modern phylogenetic systematics, despite being embedded in the title of the ICNP [13].

## Freedom within the code

As a densely written, old-fashioned, jargon-heavy, repetitive document, padded out with four prefaces and thirteen appendices, most microbiologists see the ICNP as inaccessible and intimidating. However, when stripped back to its bare essentials, the Code is surprisingly flexible—and even radical—in what it allows.

The ICNP is divided into Principles, Rules and Recommendations. The Principles, and the Rules that flow from them, have to be obeyed, whereas Recommendations can be—and often are—ignored. However, perhaps surprisingly, the Code adopts a stance that we might call *syntactic minimalism* in specifying very few linguistic Rules that have to be obeyed; almost all that it has to say on language is advisory rather than mandatory. One recent validly published name provides a striking example of how Recommendations can be ignored, so long as names comply with the Rules: *Myxococcus llanfairpwllgwyngyllgogerychwyrndrobwllllantysiliogogogochensis* [14].

So what are the Rules that have to be obeyed during the creation of genus names or species epithets? Principle 2 makes clear that bacteriologists must not use names already used for animals or plants. The Rules of the Code say that names must be treated as Latin or Latinized words. Diacritics are not allowed, nor are ordinal adjectives above ten. Genus names must be singular nouns (or adjectives used as nouns) and must be assigned a gender (masculine, feminine or neuter) and start with a capital letter. Species names are combinations of a genus name followed by a species epithet. The species epithet must be a single word in lower case and must be a noun (in the genitive case or the nominative ‘in apposition’, i.e. providing an additional description of the taxon) or an adjective that agrees in gender with the name of the genus.

And there you have it—in that single paragraph, we have specified pretty much all that the rules say you *must* do when creating names. However, our freedom to act goes even further, as the Code also adopts a stance that we might call *semantic minimalism*—which means that names do not need to be mean what they appear to say.

How does this work? Well, in some systems of nomenclature, there is a precise one-to-one relationship between the components of a word and the characteristics of the thing it is describing. For example, the rules for naming human monoclonal antibodies generate the name ‘tocilizumab’, which an expert user can decode to mean an immunomodulatory humanized monoclonal antibody targeting the cardiovascular system. By contrast, Principle 4 of the ICPN makes clear that the primary purpose of a bacterial name is simply to supply a means of referring to a taxon rather than to indicate the characters or history of the taxon. Rule 55 expands on this point by making clear that a name must not be replaced just because it does not accurately describe the taxon, citing the examples of *Bacteroides melaninogenicus*, which does not produce melanin and *Haemophilus influenzae*, which does not cause influenza. Thus, any linguistic components of a name, at most, act as an aide-memoire, rather than provide trustworthy insights into the characteristics of a taxon. This is similar to how we use personal names: for example, when someone calls herself ‘Rose Taylor’, she is not claiming to be a flower or work in the clothing industry.

The Code’s semantic minimalism goes further in that the Rules state that names can be taken from any source or may even be composed in an arbitrary manner, just so long as they are treated as Latin words. In other words, taxonomic names do not have to mean anything at all! This echoes the use of made-up personal names, such as Vanessa, or trade names, such as Häagen-Dazs, devoid of any etymology, or the use of arbitrary three-word combinations to identify places under the What3words geocode (https://what3words.com).

The linguistic minimalism inherent in the Code means that we can be as creative as we like in forming names for bacteria: it is only our community’s fusty conservatism, not the Rules, that stop us from engaging in the kind of whimsical wordplay enjoyed by the father of modern taxonomy, the Swedish naturalist Linnaeus [15], who playfully gave the blue whale the epithet *musculus,* meaning *small mouse*, and named a species of morning glory *nil*, which literally and metaphorically means *nothing*, unless one recognizes a hidden link to an Indo-Iranian root meaning blue! Similar wordplay is alive and well in contemporary zoology, with names such as *Umma gumma* and *Macrostyphlus frodo* for insects or *Ba humbugi* for a snail [16,17].

## Stability versus correctness

Aside from the linguistic freedom it grants, the Code can also be seen as a radical document in that Principle 1 opens with the words ‘Aim at stability in names’, while clarifying that the term ‘names’ refers simply ‘to scientific names applied to prokaryotes’. Similarly, Rule 61 makes clear, “The liberty of correcting a name or epithet… must be used with reserve … no grammatical or orthographic corrections will be accepted for names on the Approved Lists of Bacterial Names, the Validation Lists and the Notification Lists”.

If one takes these statements at face value, they imply that we should aim to maintain any names applied to Archaea and Bacteria in whatever context. This means that all published names, not just those validly published, should not be changed without good reason. Thus, if someone wants to overwrite an existing name, then reviewers, editors, publishers, data curators and nomenclature experts can evoke these words and discourage such a change under the authority of the Code. One might argue that interpreting the ICPN this way undercuts the need for a SeqCode, in that all names that have been published enjoy a kind of de facto priority. Furthermore, if we are to adhere to this principle, much of what passes for nomenclatural quality control—based on the Recommendations from the Code and/or subjective opinion—is rendered unnecessary and even unwelcome. So, for example, if our principal aim is stability, then why suggest changing a well-formed *Candidatus* name such as *Ca.* Rickettsia barbariae to *Ca.* Rickettsia barbarica [18], when the existing name is descriptive, well formed and has already been used in dozens of peer-reviewed publications?

The Code also brings stability in that we are not starting afresh with a blank sheet of paper but with a system of nomenclature that stretches back to Linnaeus and beyond [19]. Many aspects of Linnaean nomenclature have stood the test of time: for example, that taxonomic names are expressed in Latin and typically built from Latin and Ancient Greek roots. Aside from providing stability in practice, there are advantages to the use of Latin. As a language with no native speakers, Latin is less likely to evoke the charge of cultural imperialism than any contemporary language. Furthermore, stems from Latin and Ancient Greek are widely used in the language of science across the world so that the look-and-feel of Latinate terms often evokes feelings of familiarity, as well as gravitas.

A clear disadvantage of Latin is that few microbiologists are proficient in the grammar of the language and so make mistakes in the creation of Latin names. For those familiar with Latin, this jars on the nerves, just as spelling mistakes in English upset those of us that speak the language. However, some would argue that, as so few of us are familiar enough with Latin to be upset by grammatical errors, we should just ignore this issue and adopt an ‘approximate Latin’ approach—after all, English speakers are perfectly happy to say *museums* rather than use the Latin plural *musea*. A counter-argument is that one does not need to have enough mastery of Latin to go back in time to discuss bacteriology with Pliny the Elder, merely grasp a handful of grammatical rules and learn how to use a dictionary, which is arguably easier than, say, mastering Python or R. In addition, the ready availability of online linguistic tools and resources makes it easier than ever to exploit humanity’s common Greco-Roman linguistic heritage (Table 1).

**Table 1:** Online language resources

Link	Description
wiktionary.org	Wiktionaries in most major languages include thousands of Latin and Ancient Greek words with translations, often with etymologies, grammatical metadata and links to online dictionaries
la.wikipedia.org	Wikipedia in Latin, with many modern neologisms
la.wiktionary.org	Wiktionary in Latin
archives.nd.edu/whitaker/dictpage.htm	List of 40,000 Latin words with grammatical metadata and English translations
www.perseus.tufts.edu/hopper/search	Searchable Latin and Greek dictionaries, with an option to find Greek or Latin words based on English definitions
www.latinitium.com/latin-dictionaries	Searchable Latin dictionaries
lpsn.dsmz.de/advanced_search	Allows searches of etymologies of existing bacterial names
archive.org/details/compositionofsci00brow	Manual on the composition of scientific words, with extensive lexicon
translate.google.com	Allows translation of words from many languages into Latin and vice versa

When real or apparent linguistic errors do occur, whether in bacteriology or zoology, arguments rage as to whether stability or correctness is more important [[20], [21], [22], [23]]. The need to change well-established names on grammatical grounds clashes with the need for stability, and so some uncorrected ungrammatical names remain in use under the Code (e.g. *Bactoderma alba*, where there is disagreement in gender).

Similarly, why correct names on the basis of their meaning? Linnaeus was happy to use personal names as taxonomic names, without worrying whether this implied that the taxon and person in question were one and the same thing—e.g. whether the god *Adonis* was an ornamental plant or vice versa. By contrast, a decision in the 1990s to reject several well-established bacterial names because they might imply, say, that a bacterium is a pineapple [24,25] caused upset among those who actually used the names [[26], [27], [28], [29]]. To avoid such disputes, Rule 61 in the Code now rules out linguistic changes to names after valid publication. However, linguistic changes continue to be made in Validation Lists. Given the dwindling supply of microbiologist experts in classical languages, we need to find ways to avoid the need for linguistic revisions to names in the future (Table 2).

**Table 2:** Recent linguistic emendations and how they could be avoided

Type of change	Number	Examples	How to avoid
etymology	20	N.L. n. *petra* to Gr. fem. n. *petra*	Simpler protologues
syllabification	9	En.do.bac‘te.ri.um to En.do.bac.te’ri.um	Simpler protologues
agreement in gender	8	*aurantiacus to aurantiaca*	Online linguistic tools
change case to genitive	3	*pithecelloba to pithecellobii*	Online linguistic tools
spelling	3	*chionocetis to chionoecetis*	Online linguistic tools
geographical epithet	3	*akappagea to akappageensis*	Relaxed approach to Recommendations
connecting consonant	2	*dielmoensis to dielmonensis*	Relaxed approach to Recommendations
capitalization	2	*Xinjiangensis* to *xinjiangensis*	Online linguistic tools
connecting vowel	1	*massiliogabonensis to massiliigabonensis*	Relaxed approach to Recommendations
protologue	8	*A. facilis to Alteromonas facilis*	Simpler protologues; Online linguistic tools

Emendations were collated from Validation Lists 184-199 [[30], [31], [32], [33], [34], [35], [36], [37], [38], [39], [40], [41]].

## Making it easier to create and use names

Bacteria are given descriptive names based on phenotype (e.g. *Acidomonas*), habitat (e.g. *Enterococcus*) or phylogeny (e.g. *Alloprevotella*). They can also be named after people (e.g. *Escherichia*), places (*Massilia*, after Marseille) or organizations (e.g. *Cedecea,* after CDC). Most descriptive names are compound words built from stems recruited from Latin or Ancient Greek. Aside from specifying the need for connecting vowels, the ICNP provides scant guidance on how this should be done. By contrast, the Botanical Code makes clear that when combining nouns, one should choose stems that are derived from the form seen in the genitive case after removal of the genitive ending—so from the Latin word, *Sus, suis*, pig, we have *Suicoccus*, where the stem ‘su’ has been carefully selected and combined with the connecting vowel ‘i’ and the final word element ‘coccus’.

Such linguistic intricacies are intimidating to most microbiologists. With colleagues, I have therefore created over a million descriptive names before they are needed, using a computer program GAN that combines well-formed classical stems with relevant meanings [42]. The simplest approach was to apply prefixes to existing names, which conserves their linguistic properties while supplying a semantic hint at their phylogeny. So, you could use the name *Neoenterococcus* for a sister group to the existing genus *Enterococcus* or for a newfound coccus living in the gut, confident in the knowledge that the new name remains a masculine noun in the nominative case. Although we have not done this so far with GAN, similar use of suffixes allows for the retention of the initial used in the abbreviation of the genus name, as shown recently with *Clostridioides difficile* [43].

Combinatorial concatenation of stems provides a productive approach to generating large numbers of well-formed descriptive names that can be used off the shelf for organisms associated with a particular habitat—for example, by combining terms meaning *pig*, *gut/faeces* and *microbe* to name bacteria from the pig gut microbiome we can quickly create hundreds of new names. However, there is a trade-off here between the semantic specificity of a name and its length, as many stems with appropriate meanings are already long (e.g. *intestin-* or *excrement-*), and each additional stem makes the word even longer. Thus, a recent publication that used names created this way proposed tongue twisters such as *Hoministercoradaptatus ammoniilyticus, Anthropogastromicrobium aceti* and *Porcipelethomonas ammoniilytica* [44].

Given the Code allows the creation of names in an arbitrary manner, it seems sensible to adopt a more pragmatic approach in which elements are shortened to give more tractable names. There are already precedents for this. The genus *Demequina* has been given an arbitrary name derived from demethylmenaquinone, an unusual quinone found in this organism, while *Methermicoccus* is an arbitrary name referring to a small, thermophilic, methane-producing coccus. Reducing *Hoministercoradaptatus* to, say, *Hosterca* would certainly create a name that is easier to use. In addition, it would be easy to create software that sampled and concatenated syllables from relevant stems to create arbitrary names that retained links to descriptions but were easy to use, particularly given that a similar approach has recently been used to create genus names for viruses [45].

Another approach that could be used to create meaningful but short names would be the adoption of words from languages other than Latin or Ancient Greek—despite a Recommendation in the Code that this should only be done when no classical equivalent exists. This would open the door to short euphonious names for microbes while also adopting a more inclusive approach to non-Indo-European cultures. Thus, names for microbes associated with chicken could include *Ofimonas* (from Hebrew), *Kazabacterium* (from Hausa) or *Jicola* from Chinese.

However, given that names do not need to mean anything at all, we can go one step further and create completely arbitrary names. This approach has a long tradition in taxonomy. Linnaeus created arbitrary names via anagrams, e.g. the genus name *Mahernia* as an imperfect anagram of *Hermannia* [19]. In the 1830s, the eminent English botanist John Lindley wrote: “So impossible is it to construct generic names that will express the peculiarities of the species they represent, that I agree with those who think a good, well-sounding, unmeaning name as good as any that can be contrived” [46]. Soon after, Scottish naturalist George Johnston created the arbitrary genus name *Carinella* for a marine annelid [47]. Early 20th-century biologist William Kearfott created over a hundred arbitrary rhyming species epithets—e.g. *bana, cana, dana; bobana, cocana, dodana; boxcana, coxcana, doxcana—*many of which still belong to validly published names in use today [48]. In 1952, palaeobiologist Raymond Casey made up the Greek-sounding name *Gythemon* for an extinct clam, now cited in the Zoological Code as a name built from ‘an arbitrary combination of letters’ [49].

So, why not adopt a similar approach in bacteriology, scaled up to cope with the deluge of new species? If we do this drawing on syllables used in Latin words, we can create Latinate names that preserve the look and feel of the language (in jargon, the phonotactics of Latin) but have no meaning. To explore this option, I have taken all five-letter strings from the start of Latin words and combined them with the commonest endings from feminine nouns to create nearly 75,000 arbitrary Latinate names, which for convenience are all feminine in gender. If such names are used as genus names and as species epithets (ensuring, in the jargon, they are described as ‘nouns in apposition’), we can easily create over five billion names for bacterial species! Table 3 shows fifty species names created in this fashion. Although not all such names count as agreeable and easy to use, this simple exercise shows how arbitrary names could be created and used at scale in bacteriology while also avoiding the need for linguistic quality control.

**Table 3:** A selection of arbitrary species names

*Acentatrix varicetica*	*Adagnaria lustretica*
*Alticactea insubagena*	*Ammociquia relegellio*
*Annalutela mutularia*	*Ardifeptis rastretica*
*Arnuseptis acrufeptis*	*Aruspatrix papulutela*
*Aucilatrix merulentia*	*Batalutela scillitudo*
*Bathretica carthellio*	*Bilanadema apronilago*
*Bisaceptis rigeneptis*	*Bonasaria zizipella*
*Catulutela ascopetica*	*Comosaphia gazelagena*
*Crueneptis abnoradema*	*Cryptatrix necopitas*
*Deambia felonadema*	*Delpharisa diphricula*
*Deruparia ellebentia*	*Ditonicula ricinaphia*
*Ebiscellio arielentia*	*Ectenia ampelentia*
*Ensicactea odollascua*	*Fausarisa biosparia*
*Frusteptis prothella*	*Gasalicula ablacatrix*
*Hederarisa appulilago*	*Holocicula ulcerorea*
*Kalenatrix romphella*	*Linosella eclecatrix*
*Livoragena romphadema*	*Lubidellio furaxorea*
*Madiditas parilicula*	*Mateleptis altoritas*
*Nehaladema ulterorea*	*Nicetagena regalitudo*
*Nobiliquia aetiteptis*	*Nunciaria esculitas*
*Orestectio lapatitas*	*Positarisa absyniquia*
*Pyrenia pabulascua*	*Siruporea ophitiquia*
*Sistradema omissitudo*	*Taoractea prompaphia*
*Tetanaria visocetica*	*Triobactea venaborea*
*Venucellio avorsicula*	*Viteliquia arrogagena*

Another important issue is avoiding confusion. Principle 1, Point 2 in the Code makes clear that we must ‘avoid or reject the use of names that may cause error or confusion’. The Code specifically recommends that we avoid using similar epithets in the same genus, while Rule 56a specifies that the Judicial Commission can place ‘perplexing names’ on the list of rejected names, citing as examples potential confusion between *Bacillus limnophilus* and *Bacillus limophilus*. The ICNP provides no specific guidance on how to avoid this. However, similar problems apply in other systems of nomenclature, for example, in creating names for drugs, where the confusion between one name and another can have fatal consequences. In this case, the US Food and Drug Administration has built a solid framework for avoiding confusion [50], with software that allows users to explore the distinctiveness of any proposed name [51]. A similar approach could usefully be applied in bacteriology.

## Making it easier to name bacteria after people or places

While bacteriologists are probably happy to sacrifice semantics for usability in creating names for bacteria, they are still going to want to create names that honour people or places. However, although all such names are supposed to be created in strict accordance with the ‘rules of Latin and Latinization’, there are no universally agreed rules on how words or names from other languages are imported into Latin. For example, the Romans adopted new letters or digraphs, such as ‘y’, “ph’ or ‘ch’, to cope with sounds found in Ancient Greek but not in Latin (e.g. the close front rounded vowel in the Greek word μύκης becomes a ‘y’ in the stem *myco-* when transliterated into Latin). But the same sound in German is transliterated according to the rules of German orthography as the digraph ‘ue’, to give us *muelleri* from the surname ‘Müller’.

This illustrates the point that Latinization generally proceeds via transliteration of written characters rather than trying to approximate how words from other languages might be pronounced in Latin. However, there is often disagreement as to how languages should be written in the Roman alphabet, with several competing systems applied to Chinese, Korean, Hindi, Arabic, Hebrew, Russian, Farsi or Japanese. For example, the genus name *Sunxiuqinia* draws on the pinyin transliteration favoured in the People’s Republic of China, while the species epithet *kaohsiungensis* reflects the Wade-Giles system used in Taiwan (the epithet would be *gaoxiongensis* if transliterated via pinyin). As there is no universally agreed pronunciation for taxonomic names in Latin, those who speak no German may well render the ‘ue’ in *muelleri* as two vowel sounds, while those unfamiliar with Chinese are likely to pronounce any ‘x’ or ‘q’ in a pinyin name as they might be in Latin. What this effectively means is that there are no rules on transcription into Latin, and those coining new names from non-Latin sources should adopt whatever approach seems agreeable to them at the time.

There can be no more compelling example of overly elaborate but incomplete advice within the Code than the suggestions on how names of people should be incorporated into Latinized names for bacteria. The advice in Appendix 9 [4] focuses entirely on languages that originated in Europe, providing recommendations on how to Latinize names from Romance, Germanic, Celtic and Baltic languages while neglecting fifteen of the most commonly spoken languages: Chinese, Hindi-Urdu, Arabic, Bengali, Russian, Malay, Swahili, Punjabi, Persian, Javanese, Turkish, Tamil, Korean or Vietnamese. In addition, it pays no attention to the political implications of the advice given, for example, in telling an Irish person that their name should be Latinized as *connorius*, reflecting what they might see as oppressive Anglicization of Irish, rather than a more Gaelic alternative such as *Oconchuirus* [52]. Similarly, it neglects to consider naming systems that do not comply with the forename first, surname last convention (as in Chinese) or fail to use surnames at all (e.g. as in Iceland).

A rather absurd convention is that surnames have been treated differently according to whether they reflect ancestry (e.g. *Johnson*) or the ancestral trade of those bearing the name (e.g. *Hunter*). The former are treated as Latin family names (such as *Julius* as in *Julius Caesar*), so that during Latinization an extra ‘i’ is added to the stem, e.g. to give *Johnsonius* or *Johnsoniae*. The latter are treated as personal nicknames (*cognomina* in Latin) and do not take the extra ‘i’. In the early 1990s, MacAdoo suggested that this could be simplified by treating them all as family names, with the added ‘i’ [53]. However, the ICNP suggests a more complex solution adding the ‘i’ except where names end in ‘r’ or a vowel, where it can be used or omitted. There are plenty of examples where either approach has been adopted, e.g. *Listeria* versus *Buchnera* or *Salmonella* versus *Coxiella*.

Adding the ‘i’ to names leads to species epithets such as *johnsonii*, which are hard to pronounce*.* The Zoological Code suggest the simpler option of adding a single ‘i’ to a modern name to create a species epithet i.e. *johnsoni* instead of *johnsonii* [54]. Simplicity suggests such an approach could usefully be adopted in bacteriology.

Similar problems occur when forming compound words from stems that end in ‘i’ (including personal names Latinized with the unnecessary ‘i’), where the addition of a connecting vowel creates an internal double ‘i’. Recent expert advice in bacterial nomenclature is to follow this practice [55], even though the Ancient Romans never did this, e.g. when creating the adjective *glorificus* from the noun *gloria*. Examples of this practice in bacteriology include *Youngiibacter*, *Rumelliibacillus and Ammoniibacillus*. Adding an ‘i’ here as a connecting vowel unthinkingly draws on a rule aimed at avoiding difficult consonant clusters but instead generates a hard-to-say cluster of vowels. Simplicity suggests we should leave out the connecting vowel when it is not needed, particularly as there are plenty of precedents for using a single ‘i’, such as *Lawsonibacter, Eisenibacter* or *Ammonifex,* which comply with the Rules of the Code.

Appendix 9 [4] cites a series of neologisms—*Wigglesia* after Wigglesworth, *Stackia* after Stackebrandt or *Goodfellia* after Goodfellow—to illustrate a rather dogmatic statement “If an organism is named after a person, the name *cannot* be shortened… but *must* appear fully” (my italics). Here, it is hard to justify the modal verb *must,* when Appendix 9 lacks the force of Rules and the advice itself is undermined by precedents from botany, such as *Mecardonia* commemorating Antonio Meca y Cardonia [56], and by the bacterial name *Simkania* created to honour Simona Kahane—which is even cited elsewhere in the Appendix.

In fact, this instruction to avoid arbitrary coinages based on personal names can be turned on its head and welcomed as an attractive approach to the creation of short and simple names that allows those from diverse linguistic traditions to do as they see fit, so long as the names they produced are treated as Latin words. So, say we wanted to honour Shankar Balasubramanian, inventor of Solexa sequencing, with a genus name, it would be perfectly okay to use *Balasubria*. Similarly, the Code’s dogmatic statement “Not more than one person can be honoured in one generic name or epithet” has already been ignored in names like *Borkfalkia* (after Peer Bork and Falk Hildebrand) and *Palibaumannia* (after Paul and Linda Baumann) [57,58].

The habit of naming bacteria after places is permissible according to the ICNP and remains popular, despite the criticisms under the label of “localimania” [59,60]. However, the tone adopted in the Code’s recommendations for incorporating geographical terms into bacterial names. For example, the statement “epithets on the basis of geographical names cannot be formed as substantives in the genitive case” is used to justify changing a construct well-formed in classical Latin such as *massiliae*, meaning ‘of Marseille’—as used by Pliny the Elder—to *massiliensis,* which also means ‘of Marseille’ but in Neo-Latin. But no harm is done leaving it as *massiliae*, as has happened with the validly published name *Rickettsia massiliae*, so why force the change? As with personal names, if we allow arbitrary contractions, then the door is open to the creation of short, simple names based on geography, such as *magabonica* or *enzedica* instead of more cumbersome constructs such as *massiliigabonensis or novaezeelandiae* (or *novaezeelandense, newzealandense or aotearoaense?*).

## Minimal protologues

The Rules of the Code are refreshingly terse when it comes to mandating how a taxon must be described when first given a name. Rule 27.2 merely states that for any validly published name, there must be a description of the properties of the taxon and designation of the type, plus the derivation of the new name, but without any specification within the Code as to how this should be done. Nonetheless, over recent decades, we have seen remarkable complexity layered on top of such simplicity in the creation of elaborate and stylized ‘protologues’ (a word not used in the ICNP), complete with long descriptions and arcane linguistic components, such as syllabifications and elaborate etymologies. Falling in line with such a tradition, we incorporated etymologies into our computer program GAN [42], while a subsequent program Protologger went even further in computerizing the creation of extensive protologues [44].

But is all this really necessary? Sutcliffe and Rossello-Mora have advocated a minimalist protologue format to avoid overly long descriptions [61,62]. Similar arguments apply to the linguistic components of protologues. For a start, most bacteriologists have little or no idea how to create or interpret the syllabifications (which are not even mentioned in the Code), particularly as there is no standard pronunciation of Latin in taxonomy, and many names ignore the rules of Latin phonotactics. So, although the syllabification given for *muelleri* is muel'le.ri, how does this help anyone know how the first syllable is meant to be pronounced—with one vowel or two? Similarly, few users know or care that the pronunciation and syllabification of *Acidianu*s should end in ia'nus ‘ya-nuss’, honouring the god Janus, rather than in i'an.us ‘ee-an-uss’, more commonly seen as an adjectival ending or that a purist would insist that the ending in *Bacterioides* should be pronounced ‘oh-ee-des’ rather than ‘oy-dees’ [56]. As changes to syllabification rank as the second most common emendation in Validation Lists, much expert input could be avoided if they were simply left out of protologues while staying fully compliant with the Code.

Similarly, most etymologies are replete with detailed descriptions for each component of a word—including the language of origin, part of speech, gender, case and number. This linguistic metadata means nothing to most bacteriologists, and the choices made as to which languages are included in the evidence trail are often arbitrary. For example, the recent suggestion [31] that the etymology N.L. n. *petra* be changed to Gr. fem. n. *petra* is hard to justify when *petra* is indeed a word in Latin, used by Pliny the Elder in *Naturalis Historia* or St Jerome in his Vulgate translation of the Bible.

As corrections to etymologies rank as the commonest emendations in Validation Lists, we can echo Sutcliffe [61] in saying let us ‘rip it up and start again’ and aim for minimal descriptions of the derivations of names that leave out all the fussy details that trip people up. At a stroke, this would save time and effort in preparing protologues while also removing most of the need for linguistic curation or for programmes like Protologger. I have provided some examples of how this might work (Table 4). Such innovations should easily sit side by side with traditional practice so that no one is forced to modernize against their will when both approaches remain fully compatible with the Code.

**Table 4:** Simpler protologues

Current example	Simplified alternative
Man.gro.vi.vir'ga N.L. neut. n. *mangrovum*, a mangrove; L. fem. n. *virga*, rod; N.L. fem. n. *Mangrovivirga*, for a mangrove rod, referring to the isolation of a rod-shaped bacterium from the mangrove environment	*Mangrovivirga,* fem. n*.* a rod-shaped bacterium from mangroves; derived from roots meaning ‘mangrove’ and ‘rod’
Di.dy.mo.coc'cus Gr. masc. adj. [δίδυμος] *didymos*, pair; N.L. masc. n. *coccus*, coccus; from Gr. masc. n. [κόκκος] *kokkos*, grain, seed; N.L. masc. n. *Didymococcus*, a coccus in pairs	*Didymococcus,* masc. n*.* a coccus with cells in pairs; derived from roots meaning ‘pair’ and ‘coccus’
Af.fi.ni.bren.ne'ri.a L. masc./fem. adj. affinis, associated with, adjacent; N.L. fem. n. Brenneria, a bacterial genus; N.L. fem. n. Affinibrenneria, a genus associated with Brenneria	*Affinibrenneria,* fem. n. a genus allied to the genus *Brenneria;* derived from the existing genus name and a root meaning ‘allied to’

## Accessible, digital and inclusive

**Fig. 1:**
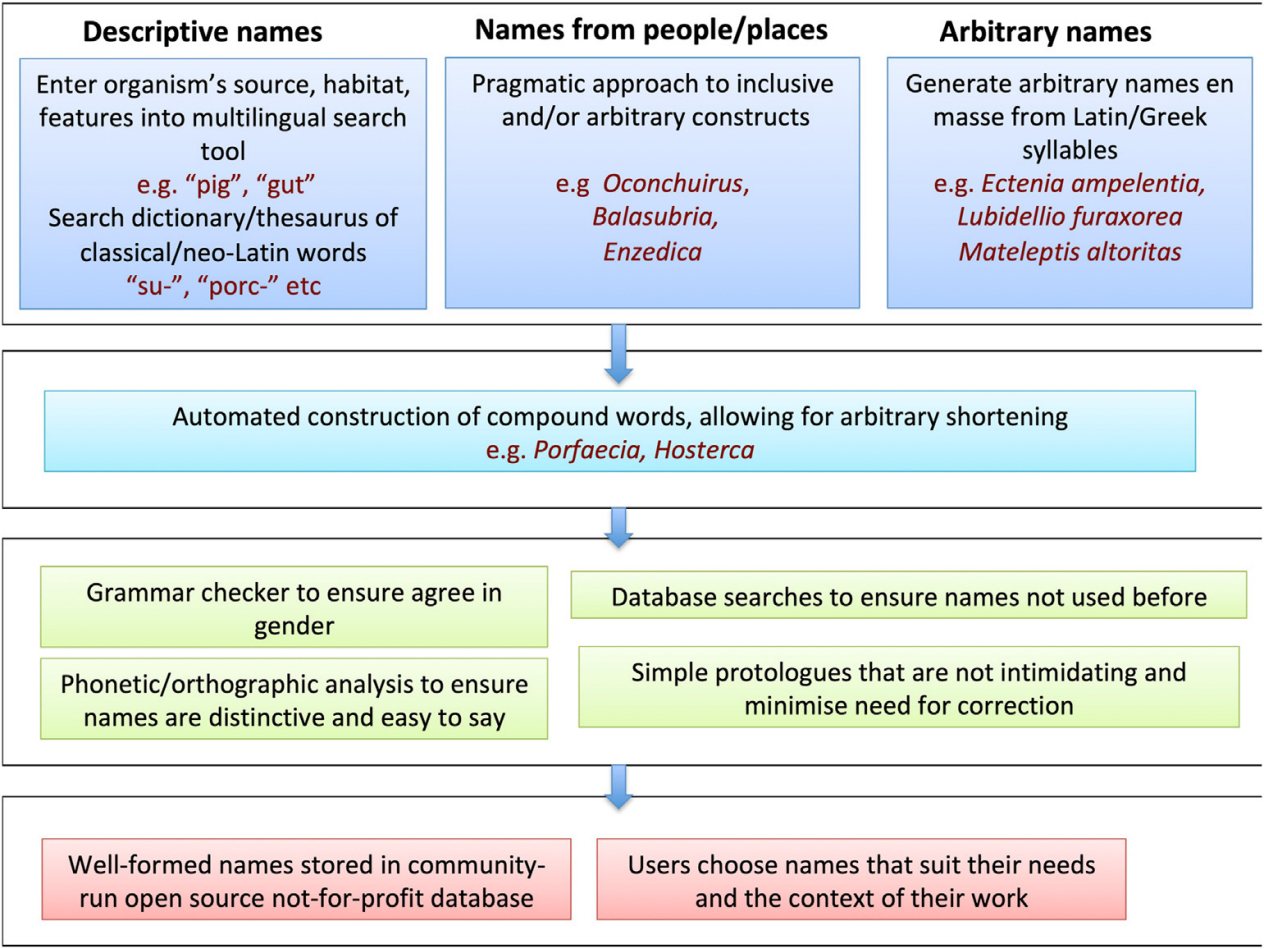
Accessible nomenclature for the era of genomics.

A key goal for coming years will be to create a user-friendly computer-based approach to the creation and curation of bacterial names that encapsulates all the points made above while making creating a name as easy as a Google search. Programs like Gan and Protologger and databases like GTDB, the LPSN [63] and the Digital Protologue Database [62] provide a promising start, but much more needs to be done to create a fully capable, comprehensive and accessible set of online resources (Fig. 1).

For automating the creation of names, such a resource might include.

• a dictionary and thesaurus of classical, neo-Latin and arbitrary word components suitable for name creation
• a search tool accepting input from common world languages to retrieve terms from the dictionary describing an organism’s source, habitat or features
• software to automate the construction of compound words
• a grammar checker to ensure agreement in gender in species names
• tools for generating arbitrary names and contractions
• tools for selecting that names are not confusing or hard to say
• tools for selecting names that have not already been used in bacteriology (including the SeqCode), zoology or botany
• an online repository of well-formed but previously unused names, where those reporting the discovery of new taxa can select choices that reflect the context in which they are working.

Our current approach to the valid publication of names through densely annotated lists harks back to an era when the only way to disseminate information was on paper or parchment. Nearly a decade ago, Rossello-Mora argued that we should move “towards a taxonomy of Bacteria and Archaea based on interactive and cumulative data repositories” [1]. Now that even the IJSEM is entirely digital [64], we should devise approaches for publishing names and associated metadata in a machine-readable format—even releasing spreadsheets in addition to or instead of lists would be a start. However, a more ambitious goal would be to create an online not-for-profit nomenclature database compliant with the expectations of open scholarship, with the regular and open release of association data, schema and software, drawing on examples such as the Digital Protologue Database, GTDB and Wikipedia. To ensure information stays up to date, those that create names should be able to deposit them directly into the database, even if subsequently subject to human or robotic oversight (cf. Wikipedia) [65]. Ideally, such a resource would become an integral part of any code and system of bacterial nomenclature, funded by learned societies or from other sources.

There is much that can be done to make our system of nomenclature more inclusive. We can learn lessons from the Swiss botanist de Candolle, who made clear in Article 2 in his 1869 *Lois de la nomenclature botanique* (ancestor to the ICNP): “The rules of nomenclature should neither be arbitrary, nor imposed by authority. They must be founded on considerations clear and forcible enough for everyone to comprehend and be disposed to accept.” [5] With that in mind, experts should adopt a non-judgmental tone, making clear that any emendation not specified in the Rules of the Code is not a correction, but just a suggestion that is open for discussion, that should be judged against the aim of providing stability and requires consent from the people who published the name. Unquestioning misinterpretation of suggestions from experts as authoritative corrections has led to the propagation of multiple names for *Candidatus* species—for example, the most common bacterial species is referred to as *Pelagibacter ubique* or *Ca.* Pelagibacter ubique in dozens of peer-reviewed publications [66], yet at the time of writing, Wikipedia insists on referring to it as *Pelagibacter communis*, simply because nomenclature experts suggested this name [58], although this emendation was not required by the Rules of the Code and despite the fact that this suggestion has not been embraced by those working on the organism.

This name provides an interesting test case in the need for linguistic pragmatism, in that although *ubique* as a species epithet fails to comply with the Rules of the Code—it is an adverb in Latin meaning ‘everywhere’, when the Code demands that species epithets be nouns or adjectives—a workaround can be found by declaring it an arbitrary noun used in apposition, inspired by the Latin word for everywhere. Such approaches are commonly used in zoology, where even an English word like *google* can be justified as a species epithet in just such a way. One might ask if almost anything is allowed within the Rules, what is to hold back creation of names like *Stalinella* or *Smellyoldsockia?* In response, Stearn, in his book *Botanical Latin* declares, “The only limitations are those imposed voluntarily by the good taste and common sense of the author”” [56]. To this, we can also add the sound judgments of journal editors and reviewers.

## Conclusions

The frantic pace of discovery of new microbial species means that this is an exhilarating time to work in microbiology. We are faced with an exciting opportunity to exploit an engaging blend of antiquity and modernity, repurposing our Greco-Latin linguistic heritage while drawing on the power of computers to cope with the deluge of new species. However, we should all agree that any system of nomenclature is a tool created by humans for communication between humans that can be changed by humans—in other words, the system should serve our research community, not the other way around.

With that in mind, future versions of the ICNP and of the SeqCode should retain the linguistic minimalism of the current Code and avoid additional layers of complexity. They should be written in a style intelligible to a wide multilingual readership and adopt inclusive language that respects the diverse identities of users. Codes should be published in multiple languages, as happens with the Botanical and Zoological Codes. Bacterial names should be easy to use, and the rules should provide minimal specifications for creating them. Most of the linguistic advice in the Code, which focuses on languages spoken in Europe and is often justified by opinion rather than argument, should be removed in future editions, particularly as it has been published elsewhere [67] and will remain available in the 2008 edition [4]. Any updated linguistic advice should be presented in peer-reviewed articles rather than within a code, and where recommendations are made, they should be justified by reasoned argument, rather than being presented as a diktat.

We have it within our power to create a revitalized code and system of nomenclature fit for the era of genomics. For the next generation of nomenclature experts, familiarity with Python or MySQL will be more important than with Latin or Greek. Let us set ourselves the challenge of naming every unnamed species in GTDB within the next year or two—after all, the competition is only a set of alphanumeric labels. We should not be forced to choose between following the rules of a long-dead language and the needs of the modern microbiology community—with a bit of creativity and pragmatism, assisted by digital tools, we can do both. The future of bacterial nomenclature is bright, bold and beautiful—but also accessible, inclusive and digital. *Semper floreat copia nominum*!

## Credit author statement

**Mark Pallen:** Conceptualization; Writing – original draft; Writing – review & editing.

## Transparency declaration

I have no conflict of interest.
